# Poly[[diaqua­(ethanol)bis­(μ_3_-pyridine-2,3-dicarboxyl­ato)dimanganese(II)] monohydrate]

**DOI:** 10.1107/S1600536809031948

**Published:** 2009-08-19

**Authors:** Yan-Qing Zhao

**Affiliations:** a Department of Chemistry, Liaoning Medical University, Jinzhou 121001, People’s Republic of China

## Abstract

The title compound, {[Mn_2_(C_7_H_3_NO_4_)_2_(C_2_H_5_OH)(H_2_O)_2_]·H_2_O}_*n*_, is a three-dimensional polymer. There are two symmetry-independent Mn^II^ centres with different coordination environments: one Mn^II^ atom is coordinated by four O atoms from four ligands and two N atoms from two ligands, the other Mn^II^ atom is coordinated by three O atoms from two ligands, two water O atoms and  the O atom of an ethanol mol­ecule. The crystal structure is stabilized by O—H⋯O hydrogen bonds.

## Related literature

For a related structure, see: Li & Li (2004 ▶). 
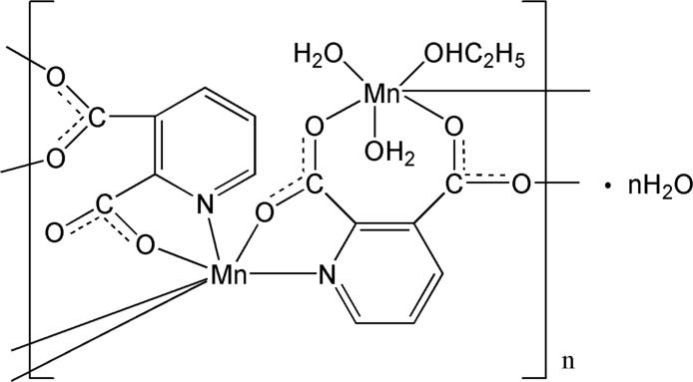


## Experimental

### 

#### Crystal data

[Mn_2_(C_7_H_3_NO_4_)_2_(C_2_H_6_O)(H_2_O)_2_]·H_2_O
*M* *_r_* = 540.20
Triclinic, 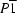

*a* = 8.4972 (3) Å
*b* = 10.2676 (4) Å
*c* = 12.6508 (4) Å
α = 72.661 (3)°
β = 74.859 (3)°
γ = 70.588 (3)°
*V* = 977.43 (6) Å^3^
*Z* = 2
Mo *K*α radiation
μ = 1.36 mm^−1^
*T* = 293 K
0.34 × 0.23 × 0.19 mm

#### Data collection

Oxford Diffraction Gemini R Ultra diffractometer
Absorption correction: multi-scan (*CrysAlis RED*; Oxford Diffraction, 2006 ▶) *T*_min_ = 0.765, *T*_max_ = 0.876 (expected range = 0.674–0.772)
11238 measured reflections
4623 independent reflections
3432 reflections with *I* > 2σ(*I*)
*R*_int_ = 0.023

#### Refinement

*R*[*F* ^2^ > 2σ(*F* ^2^)] = 0.038
*wR*(*F* ^2^) = 0.124
*S* = 1.04
4623 reflections
310 parameters
10 restraints
H atoms treated by a mixture of independent and constrained refinement
Δρ_max_ = 0.72 e Å^−3^
Δρ_min_ = −0.89 e Å^−3^

### 

Data collection: *CrysAlis CCD* (Oxford Diffraction, 2006 ▶); cell refinement: *CrysAlis CCD*; data reduction: *CrysAlis RED* (Oxford Diffraction, 2006 ▶); program(s) used to solve structure: *SHELXS97* (Sheldrick, 2008 ▶); program(s) used to refine structure: *SHELXL97* (Sheldrick, 2008 ▶); molecular graphics: *XP* (Sheldrick, 2008 ▶); software used to prepare material for publication: *SHELXL97*.

## Supplementary Material

Crystal structure: contains datablocks global, I. DOI: 10.1107/S1600536809031948/bt5015sup1.cif

Structure factors: contains datablocks I. DOI: 10.1107/S1600536809031948/bt5015Isup2.hkl

Additional supplementary materials:  crystallographic information; 3D view; checkCIF report

## Figures and Tables

**Table 1:** Hydrogen-bond geometry (Å, °)

*D*—H⋯*A*	*D*—H	H⋯*A*	*D*⋯*A*	*D*—H⋯*A*
O1*W*—H1*A*⋯O3^i^	0.827 (18)	2.29 (3)	3.083 (3)	161 (5)
O1*W*—H1*B*⋯O1	0.872 (18)	2.51 (4)	2.847 (4)	104 (3)
O1*W*—H1*B*⋯O3*W*^i^	0.872 (18)	2.63 (5)	3.034 (4)	109 (4)
O2*W*—H2*B*⋯O5^ii^	0.833 (18)	1.91 (2)	2.730 (3)	168 (5)
O3*W*—H3*B*⋯O1*W*	0.777 (16)	2.14 (2)	2.893 (4)	162 (3)
O3*W*—H3*A*⋯O6^ii^	0.793 (16)	1.99 (2)	2.699 (3)	150 (4)
O9—H9*A*⋯O8^iii^	0.821 (19)	2.10 (3)	2.776 (3)	140 (3)
O9—H9*A*⋯O4^iv^	0.821 (19)	2.319 (18)	3.006 (3)	142 (3)

Symmetry codes: (i) 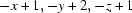; (ii) 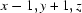; (iii) 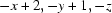; (iv) 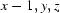.

**Fig. 1.**
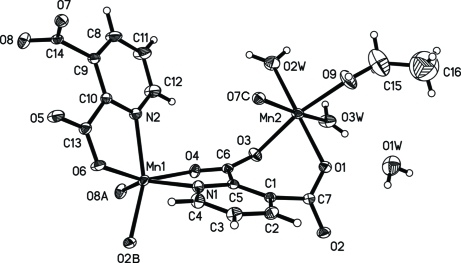
Anisotropic displacement ellipsoid (30%) plot of the title compound showing the coordination environment around the Mn atoms. [symmetry code: (A) -x + 3, -y + 1, -z; (B) -x + 2, -y + 1,-z + 1; (c) -x + 2, -y + 1, -z].

## supplementary crystallographic information

### Comment

The title compound possesses two crystallographically unique manganese cations (Fig. 1, Table 1). The Mn(1) cation :is coordinated by four oxygen atoms from four *L* ligands and two N atoms from two *L* ligands. Mn(2) cation is coordinated by three oxygen atoms from two *L* ligands, two water molecules and one ethanol molecule . The Mn—O and Mn—N distances are within the normal range observed in the structure of Li & Li (2004). In the title compound, the manganese centres are bridged by *L* ligands to form an infinite two-dimensional layer structure. Further, the water molecules and ethanol are involved in formation of hydrogen-bonding interations, leading to a three-dimensional   structure.

### Experimental

A mixture of pyridine-2,3-dicarboxylic acid (0.05 g, 0.3 mmol), MnAc_2_.4H_2_O (0.07 g, 0.3 mmol), EtOH (3 ml) and H_2_O (7 ml) was sealed in a 17 ml Teflon-lined stainless-steel cotainer. The container was heated to 140 °C and held at this temperature for 72 h. It was then cooled to room temperature at a rate of 10 °C.h^-1^. The colorless blocks   were collected in 35% yield.

### Refinement

All H atoms on C atoms were positioned geometrically and refined as riding, with C—H = 0.93 Å and *U*_iso_(H)= 1.2*U*_eq_(C). H-atoms bonded to water molecules were located in a different Fourier map and refined isotropically.

### Crystal data

**Table:**

[Mn_2_(C_7_H_3_NO_4_)_2_(C_2_H_6_O)(H_2_O)_2_]·H_2_O	*Z* = 2
*M**_r_* = 540.20	*F*(000) = 548
Triclinic, *P*1	*D*_x_ = 1.835 Mg m^−^^3^
Hall symbol: -P 1	Mo *K*α radiation, λ = 0.71073 Å
*a* = 8.4972 (3) Å	Cell parameters from 4623 reflections
*b* = 10.2676 (4) Å	θ = 1.7–29.3°
*c* = 12.6508 (4) Å	µ = 1.36 mm^−^^1^
α = 72.661 (3)°	*T* = 293 K
β = 74.859 (3)°	Block, colorless
γ = 70.588 (3)°	0.34 × 0.23 × 0.19 mm
*V* = 977.43 (6) Å^3^	

### Data collection

**Table:**

Oxford Diffraction Gemini R Ultra diffractometer	4623 independent reflections
Radiation source: fine-focus sealed tube	3432 reflections with *I* > 2σ(*I*)
graphite	*R*_int_ = 0.023
Detector resolution: 10.0 pixels mm^-1^	θ_max_ = 29.3°, θ_min_ = 1.7°
ω scans	*h* = −10→11
Absorption correction: multi-scan (*CrysAlis RED*; Oxford Diffraction, 2006)	*k* = −11→14
*T*_min_ = 0.765, *T*_max_ = 0.876	*l* = −17→16
11238 measured reflections	

### Refinement

**Table:**

Refinement on *F*^2^	Primary atom site location: structure-invariant direct methods
Least-squares matrix: full	Secondary atom site location: difference Fourier map
*R*[*F*^2^ > 2σ(*F*^2^)] = 0.038	Hydrogen site location: inferred from neighbouring sites
*wR*(*F*^2^) = 0.124	H atoms treated by a mixture of independent and constrained refinement
*S* = 1.04	*w* = 1/[σ^2^(*F*_o_^2^) + (0.0787*P*)^2^] where *P* = (*F*_o_^2^ + 2*F*_c_^2^)/3
4623 reflections	(Δ/σ)_max_ = 0.001
310 parameters	Δρ_max_ = 0.72 e Å^−^^3^
10 restraints	Δρ_min_ = −0.89 e Å^−^^3^

### Special details

**Table:**

Geometry. All e.s.d.'s (except the e.s.d. in the dihedral angle between two l.s. planes) are estimated using the full covariance matrix. The cell e.s.d.'s are taken into account individually in the estimation of e.s.d.'s in distances, angles and torsion angles; correlations between e.s.d.'s in cell parameters are only used when they are defined by crystal symmetry. An approximate (isotropic) treatment of cell e.s.d.'s is used for estimating e.s.d.'s involving l.s. planes.
Refinement. Refinement of *F*^2^ against ALL reflections. The weighted *R*-factor *wR* and goodness of fit *S* are based on *F*^2^, conventional *R*-factors *R* are based on *F*, with *F* set to zero for negative *F*^2^. The threshold expression of *F*^2^ > σ(*F*^2^) is used only for calculating *R*-factors(gt) *etc*. and is not relevant to the choice of reflections for refinement. *R*-factors based on *F*^2^ are statistically about twice as large as those based on *F*, and *R*- factors based on ALL data will be even larger.

### Fractional atomic coordinates and isotropic or equivalent isotropic displacement parameters (Å^2^)

**Table:**

	*x*	*y*	*z*	*U*_iso_*/*U*_eq_	
Mn1	1.27969 (5)	0.49235 (4)	0.24918 (3)	0.02484 (13)	
Mn2	0.59087 (5)	0.85545 (4)	0.26515 (3)	0.02779 (14)	
C1	0.7656 (3)	0.5490 (3)	0.4552 (2)	0.0231 (5)	
C2	0.7464 (4)	0.4128 (3)	0.4991 (2)	0.0308 (6)	
H2	0.6522	0.3986	0.5534	0.037*	
C3	0.8660 (4)	0.2984 (3)	0.4627 (3)	0.0336 (7)	
H3	0.8522	0.2074	0.4900	0.040*	
C4	1.0063 (4)	0.3234 (3)	0.3846 (3)	0.0336 (7)	
H4	1.0883	0.2469	0.3604	0.040*	
C5	0.9105 (3)	0.5653 (3)	0.3738 (2)	0.0228 (5)	
C6	0.9532 (3)	0.7047 (3)	0.3163 (2)	0.0230 (5)	
C7	0.6300 (3)	0.6661 (3)	0.5036 (2)	0.0247 (6)	
C8	1.2344 (5)	0.6470 (3)	−0.1599 (3)	0.0461 (9)	
H8	1.2322	0.6728	−0.2366	0.055*	
C9	1.3034 (4)	0.5069 (3)	−0.1091 (2)	0.0262 (6)	
C10	1.3014 (3)	0.4728 (3)	0.0062 (2)	0.0248 (6)	
C11	1.1694 (6)	0.7478 (4)	−0.0978 (3)	0.0575 (11)	
H11	1.1259	0.8428	−0.1317	0.069*	
C12	1.1699 (5)	0.7052 (3)	0.0170 (3)	0.0510 (10)	
H12	1.1226	0.7727	0.0600	0.061*	
C13	1.3785 (4)	0.3243 (3)	0.0709 (2)	0.0280 (6)	
C14	1.3882 (4)	0.4007 (3)	−0.1817 (2)	0.0247 (6)	
C15	0.1882 (8)	0.9931 (7)	0.2423 (7)	0.112 (2)	
H17A	0.1739	1.0126	0.1650	0.135*	
H17B	0.2214	1.0725	0.2473	0.135*	
C16	0.0088 (13)	1.0105 (12)	0.3108 (9)	0.186 (4)	
H18A	−0.0615	1.1033	0.2827	0.279*	
H18B	0.0118	0.9994	0.3884	0.279*	
H18C	−0.0368	0.9399	0.3044	0.279*	
N1	1.0296 (3)	0.4528 (2)	0.34232 (19)	0.0269 (5)	
N2	1.2358 (3)	0.5708 (2)	0.06771 (19)	0.0311 (5)	
O1	0.5328 (3)	0.7618 (2)	0.44223 (16)	0.0346 (5)	
O2	0.6140 (3)	0.6560 (2)	0.60686 (16)	0.0359 (5)	
O3	0.8368 (3)	0.8183 (2)	0.31103 (17)	0.0329 (5)	
O9	0.3290 (3)	0.8821 (3)	0.2514 (3)	0.0522 (7)	
O4	1.1066 (2)	0.6962 (2)	0.27672 (16)	0.0301 (4)	
O3W	0.5052 (4)	1.0585 (2)	0.3092 (2)	0.0479 (6)	
O5	1.4375 (3)	0.2271 (2)	0.02105 (17)	0.0443 (6)	
O6	1.3777 (3)	0.3112 (2)	0.17400 (15)	0.0346 (5)	
O1W	0.2771 (5)	0.9922 (4)	0.5189 (3)	0.0725 (9)	
O7	1.3037 (3)	0.3305 (2)	−0.19511 (16)	0.0308 (4)	
O8	1.5393 (3)	0.3966 (2)	−0.22933 (17)	0.0375 (5)	
O2W	0.6507 (4)	0.9652 (3)	0.0894 (2)	0.0520 (6)	
H9A	0.316 (3)	0.806 (3)	0.253 (4)	0.078*	
H3A	0.502 (6)	1.1229 (18)	0.2557 (19)	0.078*	
H1A	0.224 (6)	1.047 (3)	0.561 (3)	0.078*	
H1B	0.312 (6)	0.911 (3)	0.565 (3)	0.078*	
H3B	0.444 (5)	1.058 (3)	0.367 (2)	0.078*	
H2B	0.575 (4)	1.039 (3)	0.070 (3)	0.078*	
H2A	0.735 (4)	0.957 (5)	0.034 (3)	0.078*	

### Atomic displacement parameters (Å^2^)

**Table:**

	*U*^11^	*U*^22^	*U*^33^	*U*^12^	*U*^13^	*U*^23^
Mn1	0.0302 (2)	0.0226 (2)	0.0194 (2)	−0.00310 (17)	−0.00298 (15)	−0.00753 (16)
Mn2	0.0325 (2)	0.0222 (2)	0.0268 (2)	−0.00084 (18)	−0.01131 (17)	−0.00549 (17)
C1	0.0275 (13)	0.0246 (14)	0.0169 (11)	−0.0044 (11)	−0.0081 (10)	−0.0041 (10)
C2	0.0356 (16)	0.0301 (16)	0.0276 (14)	−0.0129 (13)	−0.0044 (12)	−0.0045 (12)
C3	0.0442 (18)	0.0216 (14)	0.0356 (15)	−0.0107 (13)	−0.0084 (13)	−0.0044 (12)
C4	0.0388 (17)	0.0234 (15)	0.0388 (16)	−0.0042 (12)	−0.0078 (13)	−0.0117 (12)
C5	0.0274 (13)	0.0202 (13)	0.0212 (12)	−0.0025 (10)	−0.0097 (10)	−0.0051 (10)
C6	0.0282 (14)	0.0227 (13)	0.0202 (12)	−0.0050 (11)	−0.0074 (10)	−0.0078 (10)
C7	0.0280 (14)	0.0244 (14)	0.0221 (13)	−0.0078 (11)	−0.0059 (10)	−0.0040 (11)
C8	0.071 (2)	0.0328 (18)	0.0285 (15)	0.0059 (16)	−0.0250 (15)	−0.0073 (13)
C9	0.0315 (14)	0.0242 (14)	0.0237 (13)	−0.0045 (11)	−0.0094 (11)	−0.0065 (11)
C10	0.0270 (13)	0.0227 (14)	0.0240 (13)	−0.0031 (11)	−0.0061 (10)	−0.0071 (10)
C11	0.094 (3)	0.0254 (18)	0.0385 (18)	0.0162 (18)	−0.0316 (19)	−0.0071 (15)
C12	0.080 (3)	0.0265 (17)	0.0387 (18)	0.0139 (16)	−0.0243 (17)	−0.0165 (14)
C13	0.0343 (15)	0.0239 (14)	0.0243 (13)	−0.0040 (12)	−0.0064 (11)	−0.0070 (11)
C14	0.0335 (15)	0.0207 (13)	0.0177 (12)	−0.0019 (11)	−0.0094 (10)	−0.0034 (10)
C15	0.076 (4)	0.094 (5)	0.180 (7)	−0.020 (3)	−0.040 (4)	−0.038 (5)
C16	0.156 (9)	0.191 (10)	0.215 (11)	−0.085 (8)	−0.053 (8)	0.007 (8)
N1	0.0295 (12)	0.0221 (12)	0.0276 (11)	−0.0033 (9)	−0.0053 (9)	−0.0078 (9)
N2	0.0400 (14)	0.0230 (12)	0.0258 (12)	0.0030 (10)	−0.0099 (10)	−0.0088 (10)
O1	0.0335 (11)	0.0334 (12)	0.0268 (10)	0.0020 (9)	−0.0081 (8)	−0.0024 (8)
O2	0.0414 (12)	0.0378 (12)	0.0232 (10)	0.0020 (10)	−0.0093 (8)	−0.0110 (9)
O3	0.0318 (11)	0.0210 (10)	0.0471 (12)	−0.0023 (8)	−0.0143 (9)	−0.0089 (9)
O9	0.0416 (13)	0.0287 (13)	0.0922 (19)	0.0047 (10)	−0.0303 (13)	−0.0227 (13)
O4	0.0300 (10)	0.0247 (10)	0.0336 (10)	−0.0071 (8)	0.0011 (8)	−0.0102 (8)
O3W	0.0725 (18)	0.0252 (12)	0.0428 (13)	0.0015 (12)	−0.0236 (13)	−0.0089 (10)
O5	0.0772 (17)	0.0218 (11)	0.0275 (11)	0.0061 (10)	−0.0203 (11)	−0.0094 (9)
O6	0.0564 (14)	0.0230 (10)	0.0195 (9)	0.0006 (9)	−0.0126 (9)	−0.0058 (8)
O1W	0.078 (2)	0.083 (2)	0.074 (2)	−0.0251 (19)	−0.0054 (17)	−0.0475 (18)
O7	0.0334 (11)	0.0302 (11)	0.0317 (10)	−0.0053 (9)	−0.0095 (8)	−0.0120 (9)
O8	0.0367 (12)	0.0396 (12)	0.0402 (12)	−0.0140 (10)	0.0035 (9)	−0.0199 (10)
O2W	0.0574 (16)	0.0420 (15)	0.0408 (13)	−0.0039 (12)	−0.0133 (11)	0.0058 (11)

### Geometric parameters (Å, °)

**Table:**

Mn1—O8^i^	2.127 (2)	C9—C10	1.392 (4)
Mn1—O6	2.1612 (19)	C9—C14	1.510 (4)
Mn1—O2^ii^	2.178 (2)	C10—N2	1.339 (3)
Mn1—O4	2.1874 (19)	C10—C13	1.515 (4)
Mn1—N1	2.247 (2)	C11—C12	1.386 (5)
Mn1—N2	2.281 (2)	C11—H11	0.9300
Mn2—O1	2.1542 (19)	C12—N2	1.333 (4)
Mn2—O3W	2.158 (2)	C12—H12	0.9300
Mn2—O7^iii^	2.1653 (19)	C13—O5	1.235 (3)
Mn2—O2W	2.184 (2)	C13—O6	1.269 (3)
Mn2—O3	2.195 (2)	C14—O7	1.244 (3)
Mn2—O9	2.196 (3)	C14—O8	1.260 (3)
C1—C2	1.390 (4)	C15—O9	1.353 (6)
C1—C5	1.406 (4)	C15—C16	1.526 (11)
C1—C7	1.516 (4)	C15—H17A	0.9700
C2—C3	1.382 (4)	C15—H17B	0.9700
C2—H2	0.9300	C16—H18A	0.9600
C3—C4	1.378 (4)	C16—H18B	0.9600
C3—H3	0.9300	C16—H18C	0.9600
C4—N1	1.337 (4)	O2—Mn1^ii^	2.178 (2)
C4—H4	0.9300	O9—H9A	0.821 (19)
C5—N1	1.344 (3)	O3W—H3A	0.793 (16)
C5—C6	1.515 (4)	O3W—H3B	0.777 (16)
C6—O3	1.251 (3)	O1W—H1A	0.827 (18)
C6—O4	1.252 (3)	O1W—H1B	0.872 (18)
C7—O2	1.254 (3)	O7—Mn2^iii^	2.1653 (19)
C7—O1	1.262 (3)	O8—Mn1^i^	2.127 (2)
C8—C11	1.368 (5)	O2W—H2B	0.833 (18)
C8—C9	1.383 (4)	O2W—H2A	0.863 (18)
C8—H8	0.9300		
			
O8^i^—Mn1—O6	113.83 (8)	C8—C9—C10	117.8 (2)
O8^i^—Mn1—O2^ii^	87.96 (9)	C8—C9—C14	118.9 (2)
O6—Mn1—O2^ii^	84.30 (7)	C10—C9—C14	123.1 (2)
O8^i^—Mn1—O4	80.75 (8)	N2—C10—C9	122.1 (3)
O6—Mn1—O4	155.64 (8)	N2—C10—C13	114.9 (2)
O2^ii^—Mn1—O4	116.80 (7)	C9—C10—C13	122.9 (2)
O8^i^—Mn1—N1	146.77 (8)	C8—C11—C12	118.4 (3)
O6—Mn1—N1	98.13 (9)	C8—C11—H11	120.8
O2^ii^—Mn1—N1	86.36 (8)	C12—C11—H11	120.8
O4—Mn1—N1	72.82 (8)	N2—C12—C11	122.3 (3)
O8^i^—Mn1—N2	96.47 (9)	N2—C12—H12	118.8
O6—Mn1—N2	72.70 (8)	C11—C12—H12	118.8
O2^ii^—Mn1—N2	156.42 (8)	O5—C13—O6	125.1 (3)
O4—Mn1—N2	86.78 (8)	O5—C13—C10	119.0 (2)
N1—Mn1—N2	101.68 (9)	O6—C13—C10	115.9 (2)
O1—Mn2—O3W	87.17 (9)	O7—C14—O8	125.0 (2)
O1—Mn2—O7^iii^	101.72 (8)	O7—C14—C9	118.8 (2)
O3W—Mn2—O7^iii^	170.69 (9)	O8—C14—C9	116.0 (2)
O1—Mn2—O2W	175.41 (10)	O9—C15—C16	130.6 (7)
O3W—Mn2—O2W	88.54 (10)	O9—C15—H17A	104.6
O7^iii^—Mn2—O2W	82.49 (9)	C16—C15—H17A	104.6
O1—Mn2—O3	80.79 (8)	O9—C15—H17B	104.6
O3W—Mn2—O3	89.55 (9)	C16—C15—H17B	104.6
O7^iii^—Mn2—O3	89.15 (8)	H17A—C15—H17B	105.7
O2W—Mn2—O3	97.54 (10)	C15—C16—H18A	109.5
O1—Mn2—O9	89.22 (10)	C15—C16—H18B	109.5
O3W—Mn2—O9	88.39 (10)	H18A—C16—H18B	109.5
O7^iii^—Mn2—O9	94.44 (8)	C15—C16—H18C	109.5
O2W—Mn2—O9	92.30 (11)	H18A—C16—H18C	109.5
O3—Mn2—O9	169.89 (10)	H18B—C16—H18C	109.5
C2—C1—C5	117.7 (2)	C4—N1—C5	119.6 (2)
C2—C1—C7	116.3 (2)	C4—N1—Mn1	123.72 (19)
C5—C1—C7	125.9 (2)	C5—N1—Mn1	115.42 (18)
C3—C2—C1	120.6 (3)	C12—N2—C10	119.0 (2)
C3—C2—H2	119.7	C12—N2—Mn1	125.7 (2)
C1—C2—H2	119.7	C10—N2—Mn1	114.86 (18)
C4—C3—C2	117.9 (3)	C7—O1—Mn2	128.11 (17)
C4—C3—H3	121.1	C7—O2—Mn1^ii^	138.53 (18)
C2—C3—H3	121.1	C6—O3—Mn2	125.46 (18)
N1—C4—C3	122.9 (3)	C15—O9—Mn2	134.9 (3)
N1—C4—H4	118.6	C15—O9—H9A	114.7 (19)
C3—C4—H4	118.6	Mn2—O9—H9A	110.5 (19)
N1—C5—C1	121.3 (2)	C6—O4—Mn1	119.19 (17)
N1—C5—C6	113.3 (2)	Mn2—O3W—H3A	112.5 (18)
C1—C5—C6	125.5 (2)	Mn2—O3W—H3B	112.7 (18)
O3—C6—O4	124.6 (3)	H3A—O3W—H3B	127 (3)
O3—C6—C5	119.3 (2)	C13—O6—Mn1	121.35 (17)
O4—C6—C5	116.0 (2)	H1A—O1W—H1B	103 (3)
O2—C7—O1	123.2 (2)	C14—O7—Mn2^iii^	124.82 (17)
O2—C7—C1	116.6 (2)	C14—O8—Mn1^i^	138.76 (18)
O1—C7—C1	120.0 (2)	Mn2—O2W—H2B	113 (3)
C11—C8—C9	120.3 (3)	Mn2—O2W—H2A	137 (3)
C11—C8—H8	119.9	H2B—O2W—H2A	109 (3)
C9—C8—H8	119.9		
			
C5—C1—C2—C3	−0.4 (4)	C9—C10—N2—Mn1	−172.4 (2)
C7—C1—C2—C3	−178.2 (3)	C13—C10—N2—Mn1	5.5 (3)
C1—C2—C3—C4	2.0 (5)	O8^i^—Mn1—N2—C12	−63.8 (3)
C2—C3—C4—N1	−0.9 (5)	O6—Mn1—N2—C12	−176.8 (3)
C2—C1—C5—N1	−2.4 (4)	O2^ii^—Mn1—N2—C12	−163.6 (3)
C7—C1—C5—N1	175.2 (3)	O4—Mn1—N2—C12	16.5 (3)
C2—C1—C5—C6	179.8 (2)	N1—Mn1—N2—C12	88.2 (3)
C7—C1—C5—C6	−2.6 (4)	O8^i^—Mn1—N2—C10	108.1 (2)
N1—C5—C6—O3	158.8 (2)	O6—Mn1—N2—C10	−4.9 (2)
C1—C5—C6—O3	−23.2 (4)	O2^ii^—Mn1—N2—C10	8.3 (4)
N1—C5—C6—O4	−21.0 (3)	O4—Mn1—N2—C10	−171.6 (2)
C1—C5—C6—O4	156.9 (3)	N1—Mn1—N2—C10	−99.8 (2)
C2—C1—C7—O2	61.3 (4)	O2—C7—O1—Mn2	150.7 (2)
C5—C1—C7—O2	−116.2 (3)	C1—C7—O1—Mn2	−34.5 (4)
C2—C1—C7—O1	−113.9 (3)	O3W—Mn2—O1—C7	−124.0 (3)
C5—C1—C7—O1	68.6 (4)	O7^iii^—Mn2—O1—C7	53.2 (3)
C11—C8—C9—C10	1.0 (6)	O2W—Mn2—O1—C7	−103.1 (11)
C11—C8—C9—C14	−174.5 (4)	O3—Mn2—O1—C7	−34.0 (2)
C8—C9—C10—N2	−0.1 (5)	O9—Mn2—O1—C7	147.5 (2)
C14—C9—C10—N2	175.3 (3)	O1—C7—O2—Mn1^ii^	171.5 (2)
C8—C9—C10—C13	−177.8 (3)	C1—C7—O2—Mn1^ii^	−3.5 (4)
C14—C9—C10—C13	−2.4 (4)	O4—C6—O3—Mn2	134.9 (2)
C9—C8—C11—C12	−1.9 (7)	C5—C6—O3—Mn2	−44.9 (3)
C8—C11—C12—N2	2.0 (7)	O1—Mn2—O3—C6	85.2 (2)
N2—C10—C13—O5	178.0 (3)	O3W—Mn2—O3—C6	172.4 (2)
C9—C10—C13—O5	−4.2 (5)	O7^iii^—Mn2—O3—C6	−16.8 (2)
N2—C10—C13—O6	−2.6 (4)	O2W—Mn2—O3—C6	−99.1 (2)
C9—C10—C13—O6	175.3 (3)	O9—Mn2—O3—C6	94.2 (5)
C8—C9—C14—O7	−94.3 (4)	C16—C15—O9—Mn2	−127.9 (8)
C10—C9—C14—O7	90.4 (3)	O1—Mn2—O9—C15	103.6 (6)
C8—C9—C14—O8	82.0 (4)	O3W—Mn2—O9—C15	16.4 (6)
C10—C9—C14—O8	−93.4 (3)	O7^iii^—Mn2—O9—C15	−154.7 (6)
C3—C4—N1—C5	−1.8 (4)	O2W—Mn2—O9—C15	−72.1 (6)
C3—C4—N1—Mn1	164.6 (2)	O3—Mn2—O9—C15	94.7 (7)
C1—C5—N1—C4	3.5 (4)	O3—C6—O4—Mn1	−162.1 (2)
C6—C5—N1—C4	−178.5 (2)	C5—C6—O4—Mn1	17.7 (3)
C1—C5—N1—Mn1	−164.02 (19)	O8^i^—Mn1—O4—C6	−167.4 (2)
C6—C5—N1—Mn1	14.0 (3)	O6—Mn1—O4—C6	63.3 (3)
O8^i^—Mn1—N1—C4	−132.7 (2)	O2^ii^—Mn1—O4—C6	−84.4 (2)
O6—Mn1—N1—C4	31.7 (2)	N1—Mn1—O4—C6	−7.78 (19)
O2^ii^—Mn1—N1—C4	−52.0 (2)	N2—Mn1—O4—C6	95.5 (2)
O4—Mn1—N1—C4	−171.6 (2)	O5—C13—O6—Mn1	177.4 (3)
N2—Mn1—N1—C4	105.6 (2)	C10—C13—O6—Mn1	−2.1 (3)
O8^i^—Mn1—N1—C5	34.2 (3)	O8^i^—Mn1—O6—C13	−85.8 (2)
O6—Mn1—N1—C5	−161.41 (19)	O2^ii^—Mn1—O6—C13	−171.0 (2)
O2^ii^—Mn1—N1—C5	114.9 (2)	O4—Mn1—O6—C13	37.5 (3)
O4—Mn1—N1—C5	−4.62 (18)	N1—Mn1—O6—C13	103.5 (2)
N2—Mn1—N1—C5	−87.50 (19)	N2—Mn1—O6—C13	3.7 (2)
C11—C12—N2—C10	−1.1 (6)	O8—C14—O7—Mn2^iii^	17.6 (4)
C11—C12—N2—Mn1	170.5 (3)	C9—C14—O7—Mn2^iii^	−166.56 (17)
C9—C10—N2—C12	0.2 (5)	O7—C14—O8—Mn1^i^	170.8 (2)
C13—C10—N2—C12	178.0 (3)	C9—C14—O8—Mn1^i^	−5.2 (4)

Symmetry codes: (i) −*x*+3, −*y*+1, −*z*; (ii) −*x*+2, −*y*+1, −*z*+1; (iii) −*x*+2, −*y*+1, −*z*.

### Hydrogen-bond geometry (Å, °)

**Table:**

*D*—H···*A*	*D*—H	H···*A*	*D*···*A*	*D*—H···*A*
O1W—H1A···O3^iv^	0.83 (2)	2.29 (3)	3.083 (3)	161 (5)
O1W—H1B···O1	0.87 (2)	2.51 (4)	2.847 (4)	104 (3)
O1W—H1B···O3W^iv^	0.87 (2)	2.63 (5)	3.034 (4)	109 (4)
O2W—H2B···O5^v^	0.83 (2)	1.91 (2)	2.730 (3)	168 (5)
O3W—H3B···O1W	0.78 (2)	2.14 (2)	2.893 (4)	162 (3)
O3W—H3A···O6^v^	0.79 (2)	1.99 (2)	2.699 (3)	150 (4)
O9—H9A···O8^iii^	0.82 (2)	2.10 (3)	2.776 (3)	140 (3)
O9—H9A···O4^vi^	0.82 (2)	2.32 (2)	3.006 (3)	142 (3)

Symmetry codes: (iv) −*x*+1, −*y*+2, −*z*+1; (v) *x*−1, *y*+1, *z*; (iii) −*x*+2, −*y*+1, −*z*; (vi) *x*−1, *y*, *z*.
